# *Citrus tristeza virus*: Evolution of Complex and Varied Genotypic Groups

**DOI:** 10.3389/fmicb.2013.00093

**Published:** 2013-04-23

**Authors:** S. J. Harper

**Affiliations:** ^1^Citrus Research and Education Center, Institute of Food and Agricultural Sciences, University of FloridaLake Alfred, FL, USA

**Keywords:** *Citrus tristeza virus*, evolution, strain, genotype, divergence, recombination

## Abstract

Amongst the Closteroviridae, *Citrus tristeza virus* (CTV) is almost unique in possessing a number of distinct and characterized strains, isolates of which produce a wide range of phenotype combinations among its different hosts. There is little understanding to connect genotypes to phenotypes, and to complicate matters more, these genotypes are found throughout the world as members of mixed populations within a single host plant. There is essentially no understanding of how combinations of genotypes affect symptom expression and disease severity. We know little about the evolution of the genotypes that have been characterized to date, little about the biological role of their diversity and particularly, about the effects of recombination. Additionally, genotype grouping has not been standardized. In this study we utilized an extensive array of CTV genomic information to classify the major genotypes, and to determine the major evolutionary processes that led to their formation and subsequent retention. Our analyses suggest that three major processes act on these genotypes: (1) ancestral diversification of the major CTV lineages, followed by (2) conservation and co-evolution of the major functional domains within, though not between CTV genotypes, and (3) extensive recombination between lineages that have given rise to new genotypes that have subsequently been retained within the global population. The effects of genotype diversity and host-interaction are discussed, as is a proposal for standardizing the classification of existing and novel CTV genotypes.

## Introduction

All organisms carry, in their genome, traces of their evolutionary history: past selective events, diversification, and recombination, all of which provide an insight into the adaptive landscape over which these organisms evolved. The small, simple genomes of viruses are ideal for study, as even a single non-synonymous mutation can alter the phenotype. Viral evolution and epidemiology are interdependent; the continued spread of a virus via vector species into new hosts relies on its ability to adapt (Pybus and Rambaut, 2009), although both processes are subject to drift. One aspect of viral adaptation, of any given species, is the bifurcation of an ancestral sequence or population into two or more lineages that over time develop novel phenotypic characteristics, utilize novel vectors, and infect new host species. Members of a distinct phylogenetic lineage that possess a shared evolutionary history are, to all intents and purposes, strains.

The existence of multiple strains exhibiting differences in infectivity, host range, transmission, or virulence is common amongst animal viruses, such as *Hepatitis C virus* (HCV) (Gray et al., 2011), *Influenza A virus* (Smith et al., 2009), and *Simian immunodeficiency virus* (Etienne et al., 2011), and in plant viruses, such as *Cucumber mosaic virus* (CMV) (Roossinck, 2001) and *Plum pox virus* (PPV) (Candresse and Cambra, 2006). Amongst the Closteroviridae, the existence of multiple strains is a rarity, due in part to the limited host range of most species, phloem-specific tissue tropism, or lack of opportunity for spread due to absence of polyphagous vectors (Karasev, 2000), as well as a distinct lack of research on species infecting less economically important crops. With the possible exception of *Grapevine leafroll virus-3* (Bester et al., 2012), *Citrus tristeza virus* (CTV) is the only closterovirus species to possess multiple, phylogenetically distinct strains (Moreno et al., 2008).

*Citrus tristeza virus* is one of the most significant pathogens to afflict citrus, and has been responsible for the loss of over 100 million trees either killed or rendered unproductive over the past century (Moreno et al., 2008). CTV is a member of the *Closterovirus* genus in the family *Closteroviridae*, with a 19.3-kb ssRNA genome encoding 12 open reading frames. ORF1 expresses one large polyprotein (ORF1a) containing helicase, methyltransferase, and duplicated protease domains, as well as the RNA-dependent RNA-polymerase (ORF1b) via a +1 frameshift (Karasev et al., 1995). The 10 other ORFs, expressed through subgenomic RNAs, encode the major and minor coat proteins (p25 and p27), three suppressors of RNA silencing (p25, p20, and p23) (Lu et al., 2004), two genes expressing a heat shock protein homolog (p65) and a protein with a diverged coat protein motif, both required for virion assembly (Satyanarayana et al., 2000), and three proposed host range genes (p33, p13, and p18) (Tatineni et al., 2011). CTV causes three major host reactions or syndromes: seedling yellows, stem pitting, and quick decline, of which the last two are significant problems for citrus cultivation. Symptom expression and severity is dependent on three factors: the species or cultivar infected, the species of the rootstock on which the scion is grafted, and finally, the particular infecting strain or mixture of CTV isolates (Moreno et al., 2008).

*Citrus tristeza virus* diseases, in all their forms, are the result of concentrated agricultural production; a setting quite unlike the natural environment in which both citrus and CTV evolved. Citrus have been used for trade, as a source of medicinal compounds, and as an item of religious significance for over 2000 years and have been extensively propagated throughout much of the world (Webber et al., 1967). Throughout much of their history importation of citrus plants occurred only as seed, avoiding CTV spread as the virus is not transmissible by pollen or seed (Moreno et al., 2008); it is only with the rise of rapid shipping in the mid-to-late nineteenth century that the movement of whole plants and later, live cuttings, became possible, leading to the global distribution of CTV (Moreno et al., 2008). This coincided with the rise of large-scale commercial citrus production in the late nineteenth century and adoption of monocultures; a departure from earlier production for local consumption in which a variety of species and/or cultivars were grown in one locale. Monoculture production promotes the occurrence of tristeza epidemics, which have punctuated the last century in South America in the 1930s and early 1940s, as well as Florida in 1951, Spain in 1957, Israel in 1970, and Venezuela in 1980 (Bar-Joseph et al., 1989; Moreno et al., 2008), by providing a genetically and phenotypically uniform host range susceptible to the introduction or evolution of a pathogenic strain, or combination of strains.

With the sequencing of the first CTV genomes, T36 from Florida (Karasev et al., 1995), VT from Israel (Mawassi et al., 1996), followed by T385 from Spain (Vives et al., 1999) and it’s near identical homolog T30 from Florida (Albiach-Marti et al., 2000), it became apparent that these three strains diverged markedly from one another, with two different trajectories: the VT-like and T30-like isolates on one hand, and the T36-like on the other (Hilf et al., 1999). Additional sequencing of novel isolates over the past decade suggests that the global CTV diversity is far higher than previously thought, and that new genotypes have diverged from the ancestral population, or have arisen through recombination with previously described strains (Ruiz-Ruiz et al., 2006; Harper et al., 2009, 2010; Melzer et al., 2010; Roy and Brlansky, 2010). Identification of new genotypes is complicated by asymmetry between the 5′ and 3′ halves of the genome, for most of the divergence between the groups is most apparent in the 5′ end of the genome and the ORF1a/1b genes (Hilf et al., 1999; Albiach-Marti et al., 2000) which contain the replication associated proteins. It is in the 5′ end of the genome that the more recently described T3 and NZ-B18/B165 isolates can be distinguished from one another and from VT, as they are all otherwise homologous in the 3′ subgenomic RNA coding genes (Hilf et al., 2005; Harper et al., 2009; Roy and Brlansky, 2010). Classification of CTV genotypes is further confused by the existence of recombinant isolates such SY568 (Vives et al., 2005) and HA16-5 (Melzer et al., 2010). Yet, both divergence and recombination are an important component of CTV evolution (Martin et al., 2009), and it may be proposed that the existence of multiple strains is responsible for the wide range of phenotypes observed within and between different citrus cultivars and species, particularly when multiple strains are in mixture (Scott et al., 2013).

Therefore, in this study an array of complete genomic sequences of CTV from around the world was examined to elucidate their complex and interwoven evolutionary histories, and to establish how the strains we see today came to be. Such knowledge is a necessary first step to understanding the interaction between specific virus isolates or strains and host cultivars, and hence, understanding pathogenicity. A standardized system of classification for identifying and grouping the strains present around the world, as well as a framework for incorporating novel strains, on a genotypic basis is also proposed.

## Materials and Methods

### CTV isolates

The CTV isolates examined in this study were obtained from two major sources: a collection of isolates from the state of Florida, maintained at the Citrus Research and Education Center, University of Florida, and from sequences from around the world deposited in the NCBI database (Table 1). An infectious clone based on the T36 isolate that was maintained under glasshouse conditions for 7 years in a single host was also examined (Satyanarayana et al., 1999, 2001).

**Table 1:** **Provenance of CTV isolates used in this study**.

Sequence name	Accession no.	Genotype	Isolation host	Country of origin	Sequencing method	Reference
FL202	KC517493	VT	*Citrus sinensis*	FL, USA	SOLiD 5500xl	This study
FS674	KC517485	T36	*C. sinensis*	FL, USA	SOLiD 5500xl	This study
FS701	KC517494	VT	*C. sinensis*	FL, USA	SOLiD 5500xl	This study
	KC517489	T30	
	KC517486	T36	
FS703	KC517492	VT	*C. sinensis*	FL, USA	SOLiD 5500xl	This study
	KC517491	T30	
	KC517487	T36	
FL278	KC517490	T30	*C. macrophylla*	FL, USA	SOLiD 5500xl	This study
FS577	KC517488	T36	*C. macrophylla*	FL, USA	SOLiD 5500xl	This study
FS02-2	EU937519	VT	*C. sinensis*	FL, USA	Affymetrix microarray	Weng et al. (2007)
	EU937520	T30	
	EU937521	T36	
T3	KC525952	T3	*C. sinensis*	FL, USA	Sanger	Hilf et al. (unpublished)
NZ-M16	EU857538	T3	*C. aurantifolia*	New Zealand	Sanger	Harper et al. (2009)
T68-1	JQ965169	T68	*C. sinensis*	FL, USA	Sanger	This study
HA16-5	GQ454870	Unknown	Unknown	Hawaii, USA	Sanger	Melzer et al. (2010)
NZ-B18	FJ525436	T68	*C. sinensis*	New Zealand	Sanger	Harper et al. (2009)
CT14A	JQ911663	T68	*C. sinensis*	China	Sanger	Unpublished
B165	EU076703	T68	*C. reticulata*	India	Sanger	Roy and Brlansky (2010)
NZRB-TH28	FJ525433	RB	*Poncirus trifoliata*	New Zealand	Sanger	Harper et al. (2010)
NZRB-TH30	FJ525434	RB	*Poncirus trifoliata*	New Zealand	Sanger	Harper et al. (2010)
NZRB-M17	FJ525435	RB	*C. aurantifolia*	New Zealand	Sanger	Harper et al. (2010)
NZRB-M12	FJ525431	RB	*Poncirus trifoliata*	New Zealand	Sanger	Harper et al. (2010)
NZRB-G90	FJ525432	RB	*Poncirus trifoliata*	New Zealand	Sanger	Harper et al. (2010)
B301	JF957169	RB	*C. sinensis*	Puerto Rico	Sanger	Roy et al. (unpublished)
HA18-9	GQ454869	RB	Unknown	Hawaii, USA	Sanger	Melzer et al. (2010)
T30	AY260651	T30	Unknown	FL, USA	Sanger	Albiach-Marti et al. (2000)
T385	Y18420	T30	Unknown	Spain	Sanger	Vives et al. (1999)
VT	U56902	VT	Unknown	Israel	Sanger	Mawassi et al. (1996)
T318A	DQ151548	VT	Unknown	Spain	Sanger	Ruiz-Ruiz et al. (2006)
Nuaga	AB046398	VT	Unknown	Japan	Sanger	Suastika et al. (2001)
CT11A	JQ911664	VT	*C. sinensis*	China	Sanger	Unpublished
AT-1	JQ061137	VT	*C. sinensis*	China	Sanger	Unpublished
KPG3	HM573451	VT	*C. reticulata*	India	Sanger	Biswas et al. (2012)
T36	U16304	T36	Unknown	FL, USA	Sanger	Karasev et al. (1995)
T36 (Clone)	AY170468	T36	N/A	FL, USA	Sanger	Satyanarayana et al. (2001)
538 (Clone)	N/A	T36	*C. macrophylla*	FL, USA	SOLiD 5500xl	This study

### Small RNA sequencing of CTV isolates

A total of 2 g of young green bark tissue from samples obtained either from field or glasshouse collections were ground to a fine powder in liquid nitrogen, and total RNA extracted using Trizol reagent (Invitrogen, Carlsbad, CA, USA), with modifications to the protocol to account for scale. Briefly, the powdered tissue was homogenized in 10 mL of Trizol reagent and 2 mL of chloroform and incubated on ice for 10 min. Samples were then separated by centrifugation at 12000 × *g* for 20 min, and the upper aqueous phase mixed with an equal volume of isopropanol before precipitation at −20°C for at least 2 h. Total RNA was pelleted by a further round of centrifugation, and washed with 70% ethanol before air-drying at room temperature. The pellets were re-suspended in 100 μL of dH_2_O, and the small RNA fraction, fragments of less than 200 bp, recovered by processing through an Ambion *mir*Vana miRNA isolation kit (Ambion, Austin, TX, USA) as per the manufacturer’s protocol. Small RNA presence and quality was checked on an Agilent 2100 Bioanalyzer platform (Agilent Technologies, Palo Alto, CA, USA).

Small RNA libraries were constructed using the ABI SOLiD small RNA expression kit (Applied Biosystems Inc., Foster City, CA, USA) as per the manufacturer’s protocol and sequenced using a SOLiD 5500xl platform at the Interdisciplinary Center for Biotechnology Research, University of Florida. The resulting reads for each sample were trimmed to remove adapters, and reads with a length of less than 19 nt and greater than 25 nt were discarded, giving a total of between 3.8 × 10^6^ and 1.2 × 10^7^ reads per sample. The reads for each sample were depleted by removal of sequences present in mirBase19 (Kozomara and Griffiths-Jones, 2011) and the plant snoRNA databases (Brown et al., 2003), the *Citrus sinensis* chloroplast sequence (Bausher et al., 2006), *C. sinensis* genome scaffolds, and the *Arabidopsis thaliana* mitochondrion sequence (Unseld et al., 1997). Reads for each sample were then mapped against extant genome sequences, and assembled using a combination of SHRiMP v2.0 (David et al., 2010) and CLC Genomics Workbench v5.5.1 (CLC Bio, Aarhus, Denmark), producing matches of between 9.5 × 10^5^ and 3.5 × 10^6^ reads per sequence. *De novo* assembly was also attempted using a word size of 100, length fraction of 0.5, and similarity of 0.8. Completed sequences were deposited in the NCBI database (Table 1).

### Phylogenetic and evolutionary analyses

Complete genome sequences were aligned using Muscle 3.8 (Edgar, 2004a,b) and manipulated in BioEdit 5.0.9 (Hall, 1999). Annotations were applied using CTV reference isolates T36 (Karasev et al., 1995), T30 (Albiach-Marti et al., 2000), and T318A (Ruiz-Ruiz et al., 2006) as references.

As CTV is known to frequently recombine (Vives et al., 2005; Harper et al., 2010) which creates phylogenetic ambiguity two methods, maximum parsimony (MP) and neighbor network (NN), were applied as it has been shown that these are less error prone in inferring topology in the presence of recombination (Woolley et al., 2008). MP was applied to the complete genome alignment using MEGA 5.10 (Tamura et al., 2011) with the subtree-Pruning-Regrafting algorithm with a search level of 1 in which the initial trees were obtained by the random addition of 10 sequences, branch lengths were calculated using the average pathway method. NN construction was performed using SplitsTree 4.12.3 (Huson and Bryant, 2006) with LogDet distance correction, exclusion of gap, and parsimony-uninformative sites and splits filtered using a weakly greedy algorithm.

Tests for selection, and episodic diversifying selection within sites of CTV ORFs were performed using the Fixed Effects Likelihood (FEL) (Kosakovsky Pond and Frost, 2005a) and Mixed Effects Model of Evolution (MEME) (Murrell et al., 2012) algorithms respectively, using the Datamonkey webserver (Kosakovsky Pond and Frost, 2005b). All alignments were screened for recombination, and where necessary partitioned, using the GARD algorithm (Kosakovsky Pond et al., 2006). Branch-Site Random Effects Likelihood (Branch-Site REL) analysis (Kosakovsky Pond et al., 2011) was also performed on the aforementioned alignments to search for episodic diversifying selection within branches, and for comparison with MEME results.

The presence of co-evolution between domains of ORF1a and ORF1b was detected and analyzed using the MirrorTree webserver (Ochoa and Pazos, 2010); Pearson correlation coefficient values greater than 0.8 were considered to be indicative of co-evolution (Clark et al., 2011). MatrixMatchMaker v2 (Rodinov et al., 2011) was also used to confirm co-evolutionary events within strains.

Recombination analysis was performed using RDP v3.34 (Martin et al., 2010) using the RDP (Martin and Rybicki, 2000), BootScan (Martin et al., 2005), SiScan (Gibbs et al., 2000), Chimera (Posada and Crandall, 2001), Geneconv (Padidam et al., 1999), MaxChi (Maynard Smith, 1992), and 3Seq (Boni et al., 2007) methods to generate a consensus of regions that may be recombinant in origin. Recombination events that were not identified by at least three of the seven models used were discarded, as were events for which the parental sequences could not be identified. Isolate HA16-5 was excluded from this analysis as its divergent sequence generated a large number of unconfirmed recombinant events.

## Results

### Complete genome analysis

**Figure 1:**
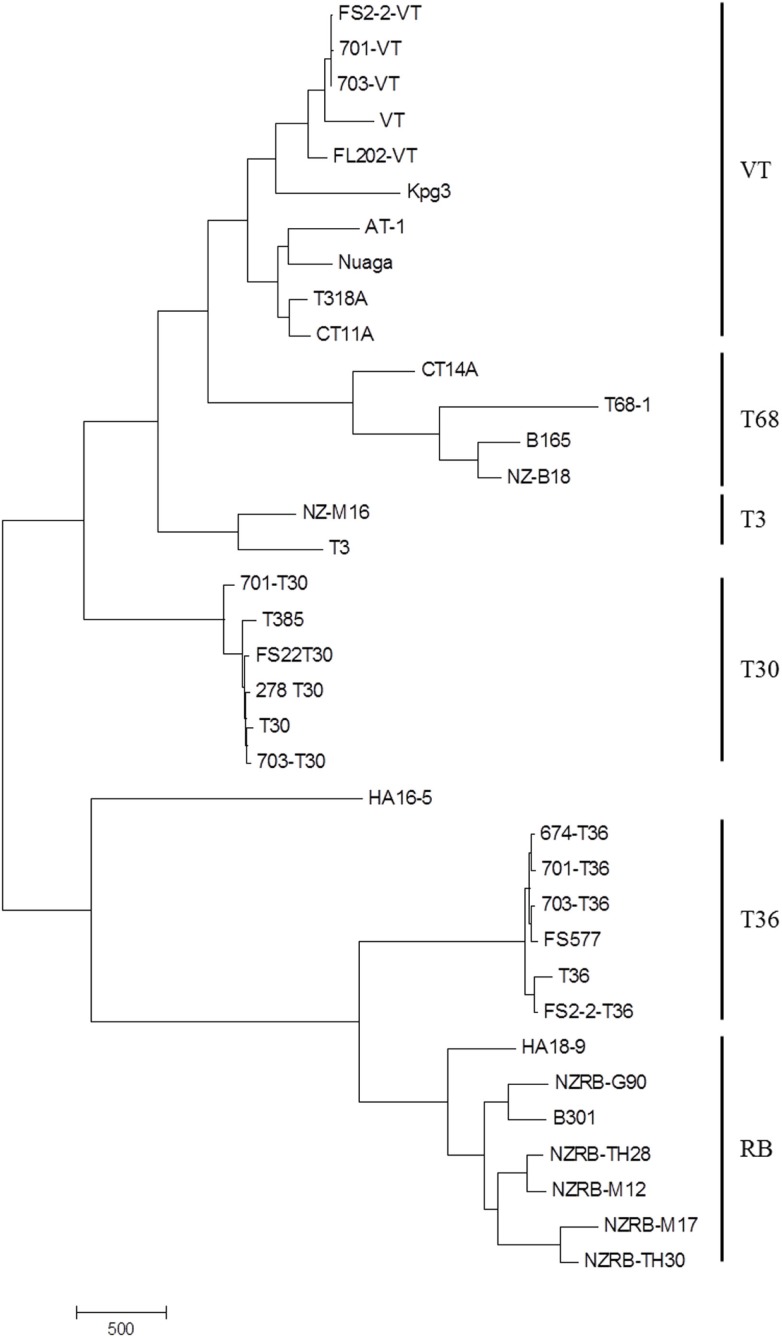
**Maximum parsimony phylogeny of the complete genomes of *Citrus tristeza virus* isolates examined in this study**. Major strains are indicated.

**Figure 2:**
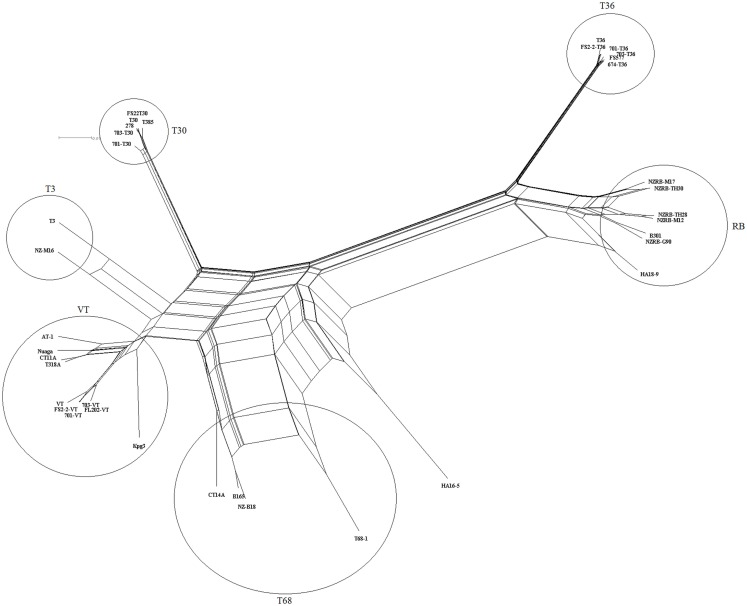
**Neighbor network reconstruction of the complete genomes of *Citrus tristeza virus* examined in this study**. Major strains groupings are indicated.

Examination of the complete genome phylogenies of 36 extant CTV sequences developed using MP (Figure 1) and NN (Figure 2) methods indicated the presence of five major previously described CTV strains: VT, T30, T3, RB, and T36. Interestingly, MP was able to resolve a further clade containing four isolates, T68-1 from Florida, CT14A from China, NZ-B18 from New Zealand, and B165 from India, that we have termed the T68 strain; NN analysis also identified this clade, though also indicated significant and repeated recombination events between this and the VT clade. This phylogeny also correctly placed the RB-T36 recombinant isolates NZRB-TH30 and NZRB-M17 (Harper et al., 2010) as part of the RB lineage rather than the T36 lineage suggested by maximum likelihood analysis (data not shown). Finally, the Hawaiian isolate HA16-5 could not be placed into one of the extant clades using MP or NN, suggesting that this is a very novel isolate and/or a recombinant as suggested by the NN analysis, and potentially a novel strain.

Bifurcation was observed within the VT lineage (Figures 1 and 2), which segregated the Israeli and US VT-like isolates, henceforth termed “Western,” from the Asian (AT-1, CT11A, and Nuaga) and Spanish (T318A) isolates, henceforth termed “Asian,” which we suggest represents the spread of two distinct sequence variants around the world, and likely reflects the historical movement of plant material. The Indian isolate KPG3, a suggested recombinant (Biswas et al., 2012), remained separate from both subtypes in the phylogeny.

Each of the major strains share an average of 85.1% nucleotide identity across the length of the genome, with a range of 92.4% nucleotide identity between VT and T3 lineages, to 80.5% between the T36 and T68 lineages (Table 2). This average identity is not evenly distributed throughout the length of the genome, for example ORF1a of the RB strain shares an average of 73.1% identity with the T30 strains, whilst the p61, p27, and p6 ORFs of these two strains possess much higher identities of 94.5, 95.5, and 95.7% respectively (Table 2 and data not shown). Amino acid identities follow a similar pattern to the nucleotide sequences, ranging between 73.4 and 92.1% for ORF1a to a high of 94.1–98.6% for p27 (Table 2). Within-strain nucleotide identities suggest conservation (Table 3), with a range of between 94.9 and 97.4% per ORF for VT and 99.2 to 99.9 for T36; T3 has lower 3′ gene identities as one member, NZ-M16, is recombinant.

**Table 2:** **Average nucleotide and amino acid between strain identities for the (A) complete genome, (B) ORF1a, (C) p25, and (D) p27 genes**.

	RB	T36	T3	T68	T30	VT	HA16-5		RB	T36	T3	T68	T30	VT	HA16-5
**(A) GENOME**								**(B) ORF1a**							
RB								RB		*91.4*	*86.9*	*92.1*	*74.8*	*74.6*	*79.6*
T36	90.9							T36	90.6		*85.7*	*91.7*	*74.6*	*74.3*	*80.2*
T3	81.0	80.5						T3	85	82.9		*85.8*	*74.4*	*74.5*	*86.1*
T68	80.9	80.5	88.0					T68	90.8	90.4	83		*74.3*	*74.3*	*79.9*
T30	82.0	81.9	90.0	86.1				T30	73.1	72.9	72.6	72.6		*91.6*	*73.5*
VT	81.2	80.8	92.4	89.9	90.3			VT	73.2	72.9	72.7	72.9	91.2		*73.4*
HA16-5	81.8	80.3	84.0	86.2	83.5	83.8		HA16-5	78.0	78.1	82.8	77.9	72.1	72.2	

	RB	T36	T3	T68	T30	VT	HA16-5		RB	T36	T3	T68	T30	VT	HA16-5

**(C) p25**								**(D) p27**							
RB		*95.7*	*96.5*	*96.8*	*95.3*	*96.4*	*97.2*	RB		*95.1*	*95.4*	*96.0*	*96.5*	*95.8*	*96.1*
T36	93.9		*95.8*	*95.6*	*96.2*	*95.2*	*96.2*	T36	92.9		*94.5*	*95.5*	*96.0*	*95.4*	*94.9*
T3	92.6	92.3		*97.8*	*96.4*	*97.4*	*98.2*	T3	87.9	87.8		*97.1*	*94.1*	*97.2*	*94.4*
T68	92.6	93.0	95.1		*95.7*	*97.9*	*97.3*	T68	89.3	89.1	92.8		*95.8*	*98.6*	*94.5*
T30	92.4	93.1	93.1	92.6		*95.5*	*96.4*	T30	95.5	93.9	88.1	89.4		*95.6*	*95.6*
VT	93.1	92.8	95.4	96.4	92.7		*97.1*	VT	89.2	89	93.8	96.2	89.5		*94.3*
HA16-5	93.4	92.2	91.9	91.1	91.5	92.4		HA16-5	92.5	92.0	87.5	88.5	92.1	88.4

*Amino acid identities are italicized*.

**Table 3:** **Average nucleotide and amino acid identities within the six major CTV strains, divided by ORF**.

	Genome		p13		p18		p20		p23		p25		p27		p61		p65		p33		p6		ORF1a		ORF1b	
	NT	AA	NT	AA	NT	AA	NT	AA	NT	AA	NT	AA	NT	AA	NT	AA	NT	AA	NT	AA	NT	AA	NT	AA	NT	AA
VT	96.4	–	94.9	96	96.1	95.4	97.4	98.6	96.3	96.8	96.3	97.5	96.1	98.5	96.2	97.2	97.3	98.3	95.3	95.6	96.3	95.8	96.2	95.9	97.4	98.5
T3	95.9	–	93.3	95	93.8	94.6	94.9	96.2	91.9	90.4	94.3	98.2	90.4	97.1	92	94.7	93.6	98.3	97.5	96	92.8	98	97.8	97.4	97.8	98.6
T68	94.2	–	96.2	95.4	97.3	98.2	98.9	99.7	98.6	97.8	98.6	99.1	98.8	99	98.1	97.7	98.2	99.2	97.8	97.3	100	100	90.8	91.8	92.2	95.3
T30	99.4	–	99.6	99.7	99.3	99.4	99.4	100	99.1	99	99.7	99.7	99.3	99.5	99.6	99.5	99.2	99	99.5	99.2	99.6	100	99.4	99.4	99.4	99.6
RB	96.2	–	94.8	93.5	95.1	94.8	95.6	97.6	95.1	94.5	96.3	97.5	95.4	97.4	96.3	95.5	97.9	98.4	96.8	95.5	97.4	96.7	95.8	95.3	97.4	98.2
T36	99.4	–	99.2	99.3	99.9	99.8	99.8	99.7	99.5	99.2	99.6	99.6	99.5	99.8	99.5	98.9	99.7	99.4	99.5	99.6	98.8	98.4	99.4	98.4	99.8	99.8

**Figure 3:**
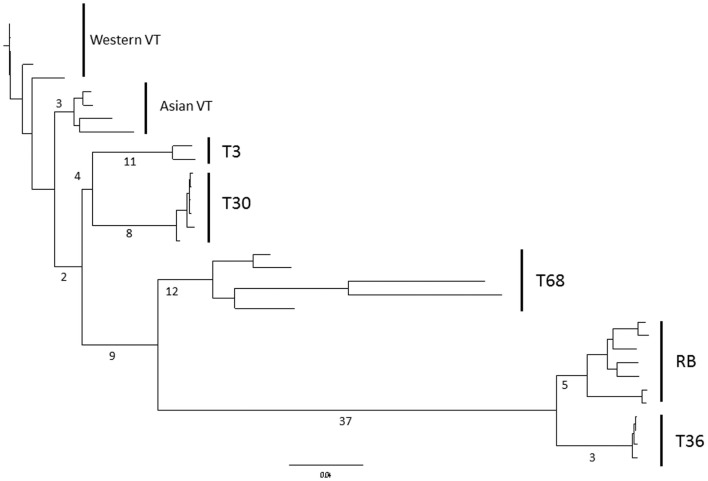
**Frequency of episodic diversifying selection events in ORF1a within CTV lineages as determined by MEME analysis, mapped on to a neighbor-joining nucleotide phylogeny**. CTV strains and subtypes indicated.

Tests for selective pressures on each of the CTV ORFs (Table 4) revealed basic patterns. First, the 3′ ORFs (p33 through p23) each have a similar proportion of codons under negative or purifying selection, ranging from 19.6 to 30.4%, while the 5′ ORFs required for replication (Karasev et al., 1995) have a much higher number of codons under negative selection with a range of 47.0–54.8%. In contrast FEL analysis, across all CTV strains, indicated that a very small proportion of codons, less than 2% in all cases, of both 5′ and 3′ ORFs show evidence of positive selection (Table 4). MEME analysis, which operates under similar assumptions, though has greater resolving power than FEL (Murrell et al., 2012), found more positively selected codons for each ORF (Table 4), of which many were selective events basal to one or more of the extant strains; the location of positively selected codons specific to single isolates rather than strains were not recorded. Even though more positively selected codons were identified by MEME, these represent less than 5% of the total which, when added to the total of negatively selected codons, suggests that the majority of the coding sequence of each ORF operates under neutral selection. In contrast to Branch-Site REL analysis which identified episodic diversifying selection only in terminal branches of VT-like isolates and indicated selection was similar between lineages (data not shown), MEME analysis did indicate significant episodic diversifying selection in sites that could be mapped to specific lineages (Table 4). This was particularly prevalent in ORFs 1a and 1b as well as p23, p33, and p61. The latter two genes possessed positively selected sites in branches leading to the RB, T30, and T36 genomes suggesting that they had, over evolutionary time, diversified from the VT, T3, and T68-like strains in these genes, while diversification in ORF1b was common to all strains except VT, with further diversification of T36; MEME did not resolve the 18 amino acid insertion unique to the T36 strain in ORF1b. Mapping the number of events onto a neighbor-joining phylogeny of ORF1a (Figure 3) revealed that there has been significant episodic diversification in first the T36, RB, and T68 lineages from T3, VT, and T30 (9 events), followed by separation of RB and T36 from the T68 lineage (37 and 12 events), and RB from T36 (5 and 3 events respectively). There are also a large number of positively selected sites, 11 and 8 respectively, in the bifurcation of the T3 and T30 genotypes, and three sites under selection in the branch leading to the Asian VT isolates, separating them from the Western VT isolates. These data therefore suggest significant, concerted separation of the major CTV lineages, and it should be noted the analysis likely underestimates the total number of diversifying events as negative selection in extant isolates to maintain sequence can obscure ancestral positive selection (Murrell et al., 2012), as suggested by Branch-Site REL analysis in this study (data not shown).

**Table 4:** **Positively and negatively selected codons present in CTV ORFs identified by FEL and MEME analysis**.

CTV gene		Fixed effects likelihood model		Mixed effects model of evolution	
ORF	No. codons	No. negatively selected sites	No. positively selected sites	Sites with episodic diversifying selection	Lineage specific codon diversification
p6	51	15 (29.4%)	0	0	
p13	119	36 (29.4%)	1 (0.8%)	1 (0.8%)	6 (RB, T36)
p18	167	41 (24.5%)	2 (1.7%)	2 (1.7%)	
p20	182	42 (23.1%)	0	4 (2.2%)	105 (Florida VT); 115 (T3)
p23	209	41 (19.6%)	4 (1.9%)	8 (3.8%)	3 (T36); 27 (T36) (RB, T30); 29 (RB, T30); 78 (RB, T30); 79 (T36) (RB, T30);177 (RB)
p25	223	57 (25.5%)	0	4 (1.8%)	
p27	240	73 (30.4%)	1 (0.4%)	2 (0.8%)	102 (T36)
p33	303	84 (27.7%)	2 (0.7%)	8 (2.4%)	117 (VT, T68, and T3); 219 (T68); 224 (Florida VT)
p61	536	137 (25.6%)	8 (1.5%)	17 (3.2%)	203 (T30); 333 (RB, T30, and T36); 372 (RB, T30, and T36)
p65	594	178 (29.9%)	3 (0.5%)	13 (2.2%)	412 (T30)
ORF1b	500	274 (54.8%)	5 (1.0%)	11 (2.2%)	237 (RB and T36)
ORF1a	3124	1469 (47.0%)	59 (1.8%)	138 (4.4%)	7 (T3, RB, T30, T36, T68); 27 (T36, RB); 30 (T68); 39 (T68, T36, RB); 47 (T36, RB); 49 (T36, RB); 52 (T36, RB); 53 (T36, RB) (Asian VT); 62 (T36, RB); 75 (T36, RB, T68); 92 (RB); 95 (T68) (T3, T30); 109 (T30) (T36, RB); 163 (T68, RB, T36); 179 (T3, T30) (T68, RB, T36); 234 (T3, T30) (RB); 238 (T3); 255 (T68, RB, T36); 299 (T3) (T68, RB, T36); 302 (RB) (T68); 310 (RB, T36); 336 (RB, T36); 378 (T30); 442 (T68); 471 (T68); 505 (RB, T36); 548 (T68); 571 (T68); 595 (RB, T36); 660 (T68, RB, T36);661 (T36) (T68); 671 (RB, T36); 694 (RB, T36, T68) (T3); 698 (RB, T36) (T68); 699 (RB, T36) (T3) (T68); 718 (T36, RB); 762 (T36, RB); 767 (T68); 875 (RB, T36); 881 (T68, RB, T36); 884 (T68, T3); 924 (RB); 1003 (T30) (RB, T36); 1075 (T68, RB, T36); 1527 (T3) (T36, RB); 1596 (T36); 1607 (T3, Asian VT); 1660 (T3); 1671 (T3), (T36, RB); 1845 (RB, T36); 1850 (T36); 1995 (RB, T36); 2004 (T30); 2016 (RB, T36); 2026 (T3), (T36, RB); 2030 (Asian VT) (T36, RB); 2039 (RB, T36); 2043 (RB); 2073 (Asian VT) (T3) (T36, RB); 2093 (RB, T36); 2103 (RB, T36); 2125 (RB, T36); 2129 (RB, T36); 2143 (T30) (RB, T36); 2168 (T3); 2173 (T30, T36); 2329 (T30); 2381 (T30); 2384 (T3, T30); 2397 (T30) (RB, T36); 2428 (RB, T36); 2464 (T30); 2526 (RB, T36); 2656 (T36, RB); 2683 (RB, T36); 2785 (RB, T36); 2852 (T68)

*Location of strain-specific codon diversification given, per ORF*.

ORF1a is an example of the varying selective pressures within a single gene. It contains four domains: the L1 and L2 papain-like proteases, methyltransferase, and helicase domains (Karasev et al., 1995), separated by regions that if not non-coding, are of unknown function at time of writing. The four domains all show conservation of sequence; FEL analysis identified between 43.2 and 66.2% of residues under negative selection, and between 0 and 1.4% of residues under positive selection, higher than the surrounding regions which ranged between 33.1 and 63.9% and 1.9 and 12.5% for negative and positively selected residues respectively (Table 5). MEME analysis detected more positively selected codons, although several of these pertained to single isolates rather than historical evolutionary events during strain evolution (data not shown). This strong negative selective pressure is reflected in the overall level of amino acid identity in all four of the functional domains, ranging between 86.2 and 93.6% for the L2 protease and methyltransferase respectively, while being notably lower between domains, with an amino acid identity range of 74.7–89.2%.

**Table 5:** **Positively and negatively selected codons present within recognized domains of ORF1a identified by FEL and MEME analysis**.

Domain	Sites	Amino acid identity (%)	No. residues	No. negatively selected sites	No. positively selected sites	
					FEL	MEME
L1	1–338	82.5	338	112 (33.1%)	14 (4.1%)	36 (10.7%)
	339–485	91.3	146	63 (43.2%)	2 (1.4%)	6 (4.1%)
	486–831	74.7	345	132 (38.3%)	11 (3.2%)	30 (8.7%)
L2	832–977	86.2	145	79 (54.5%)	0	7 (4.8%)
	978–1039	89.2	61	39 (63.9%)	0	1 (1.6%)
MET	1040–1349	93.6	309	174 (56.3%)	1 (0.3%)	4 (1.3%)
	1350–2701	79.8	1351	609 (45.1%)	26 (1.9%)	71 (5.3%)
HEL	2702–3099	92.2	397	263 (66.2%)	3 (0.8%)	7 (1.8%)
	3100–3124	83.3	24	0	3 (12.5%)	4 (16.7%)

Co-evolution was detected using MirrorTree between the ORF1a and ORF1b (RdRp) domains L1-L2, L1-MET, L1-HEL, L1-RdRp, L2-MET, L2-HEL, L2-RdRp, MET-HEL, MET-RdRp, and HEL-RdRp with Pearson’s correlation coefficient values of between 0.847 and 0.972 (Table 6). Higher coefficient values were obtained within strains for each of these events, for example within L1-MET the coefficient values were 0.919, 0.994, and 0.942 between isolates of the T30, VT, RB, and T36, strains respectively; the latter two strains share the same motifs and were considered together. This was not consistent across all domains examined, however, as some pairings only one strain had a coefficient value above the threshold, such as L2-HEL and MET-HEL in which the RB/T36 strain had values of 0.971 and 0.976 respectively (Table 6). In contrast, the MatrixMatchMaker algorithm found only weak evidence of co-evolution in most domains with weighted scores of less than 1, with the exception of VT isolates between L1 and L2, and VT and T3 isolates between the L2-HEL domains (data not shown). This is to be expected as MMM is not optimized for resolving co-evolution between closely related domains (Clark et al., 2011). Overall, these results correlate with the translated amino acid sequence of four domains of ORF1a, in which the major genotypes maintain a unique motif of amino acid substitutions, suggesting that co-evolution has occurred not only between domains, but have co-evolved within strains.

**Table 6:** **Strain-specific co-evolution events between recognized domains of ORF1a identified by MirrorTree**.

	L1		L2		MET		HEL	
L2	Overall	0.862						
	**RB and T36**	**0.974**						
	**T30**	**0.901**						
	VT	0.184						
MET	Overall	0.884	Overall	0.89				
	**RB and T36**	**0.942**	**RB and T36**	**0.977**				
	**T30**	**0.919**	T30	0.111				
	**VT**	**0.994**	**VT**	**0.941**				
HEL	Overall	0.847	Overall	0.972	Overall	0.927		
	**RB and T36**	**0.936**	**RB and T36**	**0.971**	**RB and T36**	**0.976**		
	T30	0.732	T30	0.722	T30	0.45		
	**VT**	**0.986**	VT	−0.196	VT	0.751		
RDRP	Overall	0.865	Overall	0.765	Overall	0.92	Overall	0.832
	RB and T36	0	RB and T36	0	RB and T36	0	RB and T36	0
	**T30**	**0.962**	T30	−0.397	T30	0.668	**T30**	**0.896**
	**VT**	**0.992**	VT	−0.556	**VT**	**0.967**	**VT**	**0.992**

*Events with a Pearson’s correlation coefficient of 0.8 or higher were considered to be significant (highlighted in bold)*.

### Recombination analysis

**Figure 4:**
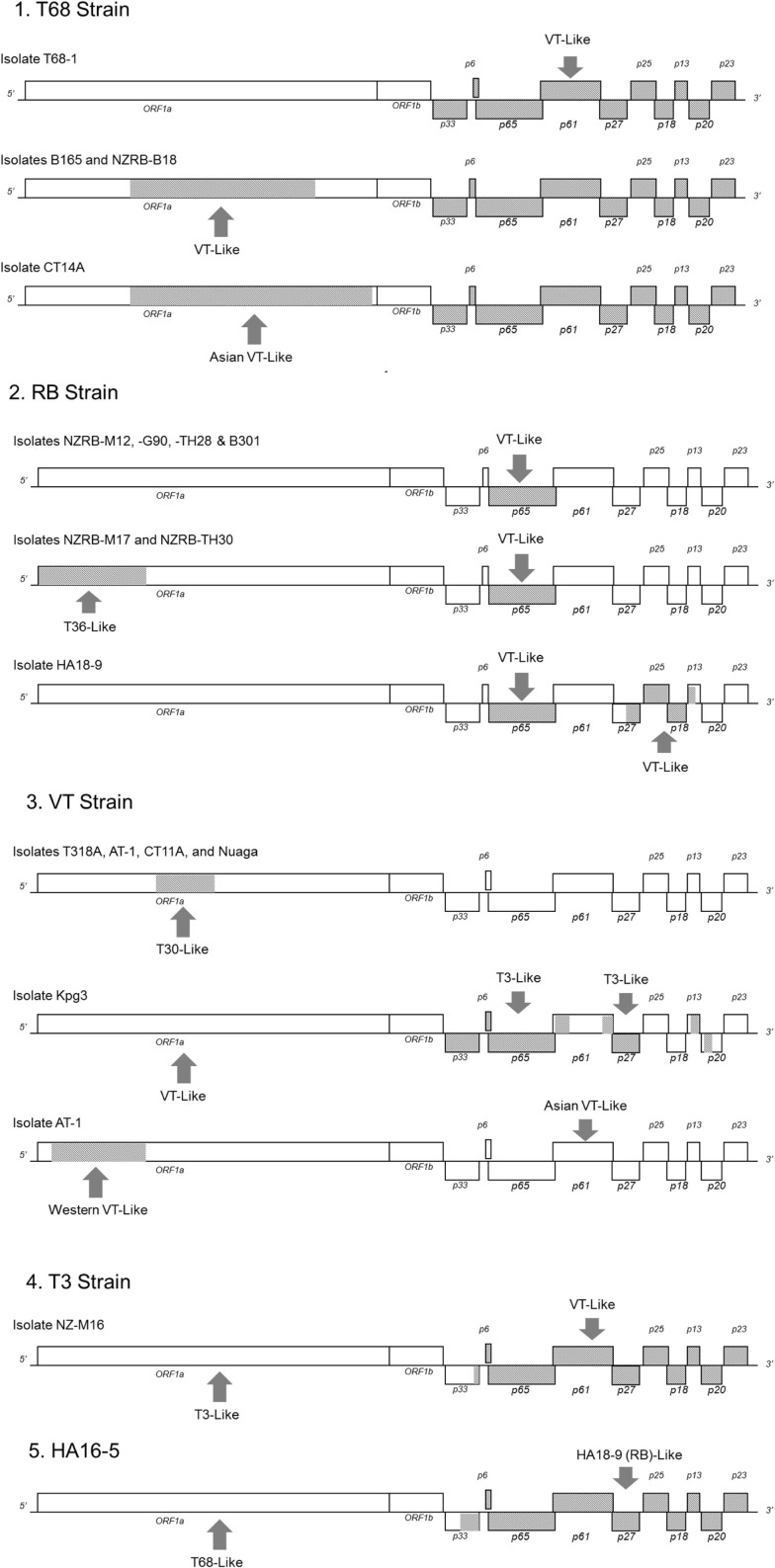
**Location and origin of the recombination events present in CTV isolates examined in this study, divided by strain**.

Recombination is a major factor in the evolution of the recognized CTV strains as indicated by the NN analysis (Figure 2). Analysis of the extant genome sequences in this study using RDP found that nearly every isolate contained trace evidence of recombination either within or between strains, although these events were weakly supported and identified by less than four models, or the parental sequences could not be identified. Recombination events supported by four or more models, with acceptable *p*-values (*p* < 0.01), were identified in members of four strains, RB, VT, T3, and T68 as well as the potentially novel strain HA16-5 (Table 7), and can be classified into two major groupings: the insertion of fragments within an ORF, or the complete replacement of the 3′ or 5′ half the genome at a point within or between the ORF1b and p33 ORFs (Figure 4). The former includes both inter- and intra-strain recombination, for example members of the RB all retain an ancestral recombination event, the partial replacement of the p65 ORF from a VT-like isolate, while three isolates also have undergone subsequent recombination events, with NZRB-M17 and TH30 of the RB strain acquiring T36-like segments at the beginning of ORF1a, while HA18-9 has acquired a VT-like segment between the partial p27 through partial p13 ORFs (Table 7; Figure 4). Three of the four T68 isolates have acquired VT-like fragments in ORF1a, although interestingly while isolates B165 and NZ-B18 possess the western VT-like insertions, isolate CT14A maintains a longer ∼5 kb fragment that shares higher identity to Asian VT-like isolates of 97.3 versus 94.7% to the western VT isolates. The VT-like isolates by contrast show only two events of inter-strain recombination, with a T30-like insertion in ORF1a between bases 4368 and 5695, and repeated T3-like insertions in the 3′ half of isolate Kpg3 (Figure 4). Finally, isolate AT-1, an Asian VT-like isolate maintains an insertion of approximately 3.1 kb that shares higher identity with western VT isolates; it cannot be discounted that this is the result of conservation of an ancestral proto-VT sequence rather than recombination.

**Table 7:** **Location and provenance of recombination events present in CTV isolates examined in this study**.

Recombinant isolate	Start	End	Parental strain 1	Parental strain 2	RDP	Geneconv	BootScan	MaxChi	Chimera	SiSscan	3Seq
M17/TH30	111	3281	T36	RB	5.64E-47	6.46E-24		6.92E-29	1.09E-31	7.88E-29	1.47E-81
B165, NZ-B18	3949	8305	VTs	T68	1.25E-05			3.18E-04	8.65E-06		2.72E-40
CT14A	5229	9390	Asian VTs	T68	1.98E-210	7.77E-197	1.29E-102	1.63E-68	2.91E-68	2.63E-90	1.55E-316
B165	607	1012	VTs	T68	2.33E-56	7.77E-54		1.40E-15	5.33E-15	6.40E-15	3.66E-27
AT-1	393	3489	VTs	Asian VTs	3.22E-25	1.70E-30	1.33E-16	9.30E-24	3.59E-17	3.38E-14	6.22E-49
RB	12175	13809	VTs	T30	1.69E-14		9.47E-14	2.07E-14	4.07E-16	6.78E-17	9.21E-26
NZ-B18	13154	13605	RB	T68	6.46E-18	5.55E-16	9.67E-12	6.39E-05	6.17E-05	3.21E-04	
CT14A	16667	17410	VT	T3	7.75E-12	1.21E-07	4.13E-07	1.20E-04	2.65E-05	2.56E-04	
NZ-M16	36	11532	VT	T3	2.82E-98	1.99E-85	7.43E-88	4.78E-40	8.65E-05	2.10E-43	5.70E-158
HA16-1	21	11281	HA18-9	T68-1	1.84E-317	3.84E-303		3.43E-42	1.12E-54	9.18E-74	5.78E-218
Asian VTs	4368	5695	T30	VTs	6.60E-04		1.25E-02	9.71E-08	4.29E-08	2.81E-08	2.20E-02
T68-1	7	10848	Unknown (itself?)	VTs	7.44E-160	7.08E-90	2.17E-66	1.39E-46	4.01E-46	1.00E-34	6.53E-168
Kpg3	10804	14424	T3	VTs	4.31E-52	6.31E-37	1.17E-37	5.04E-27	6.46E-29	6.67E-29	4.56E-75
Kpg3	14858	16000	T3	VTs	8.65E-24	2.50E-16	3.28E-26	1.10E-09	1.47E-09	2.35E-11	3.30E-27
Kpg3	17438	18040	T3	VTs	1.61E-08	2.03E-03	7.09E-09			6.19E-03	
HA18-9	15781	17434	VT	NZRB-TH28	1.63E-85	7.03E-64	2.66E-86	1.87E-13	8.29E-08	4.25E-18	4.61E-26

The replacement of the 5′ or 3′ half of the genome also occurs, most notably in the unclassified isolate HA16-5 which possesses a complete 3′ replacement, introducing an RB, or more specifically HA18-9 fragment, on to the end of a T68-like ORF1, while NZ-M16, a T3-like isolate has a VT-like complete 3′ replacement. All members of the T68 strain possess a complete 3′ replacement with a VT-like isolate that is likely the result of a single ancestral event, as it is largely conserved between T68-like isolates with an average 98.3% nucleotide identity.

### Evolution within lineages

It has already been observed that there is a high degree of similarity within but not between strains; in this study two lineages, VT and T36, were examined in detail for evidence of within-strain evolution to determine how and where closely related isolates diverge from one another. The VT strain is, at present, the most diverse of the recognized strains, with members sharing an average of 96.4% nucleotide identity (Table 2). As mentioned earlier, the VT strain can be separated into two sub-strains, encompassing the Israeli and US VTs, and the Spanish and Asian VTs; the VT-like Kpg3 isolate is a recombinant and does not group with the two major clades. The two subgroups differ by 3.7% at the nucleotide level, with the majority of the diversification located in ORF1a. Although it should be noted that comparatively few result in positively selected non-synonymous substitutions, with only six in ORF1a, one in p20 and two in p61 (data not shown). Most show no evidence of positive or negative selection and simply may be neutral for strain evolution. Despite diversification, it should also be noted that both subgroups contain the conserved VT-strain motifs in the ORF1a L1, L2, MET, and HEL domains; and as mentioned earlier, show evidence of within-strain co-evolution between these domains.

The T36 strain shows significantly less diversity, based on extant sequences, than the VT strain with an average nucleotide identity of 99.4% (Table 2), which may be due in part to the sequencing of isolates from one geographic locale, and no obvious segregation into sub-strains. There is a small divergence of 0.46–0.57% at the nucleotide level between the type isolate of the strain, T36, which has been propagated in glasshouse conditions for approximately 40 years (S. Garnsey, personal communication), and isolates FS577, FS674, and FS701 extracted recently from field samples. These minor changes are significant in that the T36 isolate is phenotypically different from the extant field isolates, with a decrease in aphid transmission efficiency from approximately 40–50% down to 1% by *Toxoptera citricida*, and a decrease in virulence, producing only mild stem pitting on susceptible *C. macrophylla* hosts (S. Garnsey, personal communication; Harper, unpublished). The substitutions are distributed throughout the genome and have produced a total of 17 non-synonymous mutations in ORF1a, nine in p61, three in p65, and one change each in p6, p18, p20, p23, p25, and p33.

Finally, the possession of a T36 based clone allowed us to explore the evolutionary rate of a single isolate. Isolate 538, introduced by bark-flap inoculation of a virion preparation into a *C. macrophylla* host 7 years earlier, was reconstructed by sequencing of the siRNA population present. Comparison of the reconstructed isolate 538 sequence with the clone reference sequence AY170468 indicated that only nine nucleotide substitutions had become fixed in the consensus sequence, an evolutionary rate of 6.67 × 10^−5^ per site, per year; these substitutions included five non-synonymous substitutions, located in ORF1a (positions 606 T-A and 2228 T-I), p61 (324 G-D), and p18 (59 I-V, and 129 K-M). Two of the substitutions (p61 324 G-D, and p18 129 K-M) restored the residue to that found in the T36-like field samples, while the others introduced amino acids of similar properties, with the exception of the substitution at site 2228 of ORF1a, which may be the result of drift or neutral evolution. This indicates a very slow rate of evolution in a single CTV isolate, under stable conditions, over time.

## Discussion

Before considering how the extant strains of CTV evolved, we should ask a more basic question: what is a strain in this context? Throughout much of their history, CTV isolates were classified by the presence or absence, and severity of, symptoms on citrus indicator species, and later by serological classification using monoclonal antibodies, such as MCA13, that distinguished between mild and severe strains (Moreno et al., 2008). It is only with the advent of sequencing, over the past quarter century, that strain classification was applied on a genetic basis.

In this study we apply the label “strain” to describe a single phylogenetic lineage, which implies a high level of sequence identity and a shared evolutionary history. It is important to reiterate here that one cannot apply a phenotypic label, such as a “seedling yellows” or a “stem pitting” isolate on a genetic basis alone. Phylogenetic analysis indicated the existence of at least six extant strains, named T36, VT, T3, RB, T68, and T30; the recombinant isolate HA16-5 (Melzer et al., 2010) represents a potential seventh strain, although until homologs are found this remains speculative. An examination of genomes of these six strains indicates that their evolutionary history is a complex mixture of diversification, with differential selective pressures operating within and between genes, as well as between strains, of extensive recombination, and adaptation to an ever changing environment. How this process occurred is described in the following discussion.

### The evolution of *Citrus tristeza virus* strains

The adaptive landscape, first proposed by Wright (1932) is a means of projecting all possible mutations and gene combinations of a species or population onto a topography on which selective pressures from the environment create fitness peaks and valleys (Wright, 1988; Pigliucci, 2008). The combined processes of mutation, selection, and drift drive a species or population across this landscape. In essence, to explore the landscape is to evolve. If we apply this metaphor to CTV, can we reconstruct the evolutionary history, the processes and selective pressures that have produced the six extant CTV lineages?

First, we must consider whether there was either a single, common ancestral proto-CTV sequence that has diversified, or whether there were multiple introductions of a proto-closterovirus into citrus. Evidence for the latter is subject to conjecture as only the 5′ half of the genome supports this hypothesis, due to the conservation of sequence in the 3′ half of the genome (Mawassi et al., 1996; Hilf et al., 1999). It has been proposed that this asymmetry results from the recombination between a proto-CTV isolate and an unknown closterovirus (Karasev, 2000), which is plausible as recombination between different virus species or families has been observed in both animal (Maori et al., 2007; Davidson and Silva, 2008) and plant viruses (Fernandez-Cuartero et al., 1994; Tan et al., 2004; Tiendrebeogo et al., 2012), and is particularly common amongst luteoviridis (Gibbs and Cooper, 1995; Smith et al., 2000). Recombination, particularly between species, allows a distinct shift in evolutionary trajectory (Sztuba-Solinska et al., 2011), moving the sequence across the adaptive landscape. Such shifts cannot occur by stepwise mutation alone, unless the selective constraints are relaxed, for stabilizing selection will tend to keep a population grouped around an adaptive peak, where any non-neutral mutant is likely to have lower fitness, and to shift between peaks will require multiple mutations to pass through a “valley” of lower fitness (Wright, 1988; Pigliucci, 2008), a cost avoided by recombination.

The extant recombinant CTV sequences HA16-5 (Melzer et al., 2010) and NZ-M16, as well as the T68 and RB strains (Harper et al., 2010), indicate that 5′-3′ recombination events are common, and the ORF1b-p33 junction may represent a selectively favored site for recombination as has been observed in other virus species (Smith et al., 2000; Ohshima et al., 2007). If we consider VT, T3, and T30, which share 90.6% nucleotide identity in ORF1a to be descendants of one proto-CTV, this suggests that there were two additional proto-CTVs or unknown closteroviruses introduced into citrus, whose descendants are T36 and RB, and T68 and HA16-5 respectively. It is also possible that the strain-specific divergence of ORF1a may be the recombination of the proto-CTV with a CTV-derived defective RNA (dRNA), as dRNAs have been proposed to act as “spare parts” to repair, via recombination, mutated, or non-functional genomic sequences (Batuman et al., 2010). dRNAs are frequently found in mixture with intact CTV isolates (Mawassi et al., 1995; Ayllon et al., 1999) and as they are non-coding and replicated by a helper genome, have the potential to diverge from the parental sequence under neutral selective conditions. Yet evidence for divergence, or eventual mutational meltdown and elimination via Muller’s ratchet, is lacking as most CTV dRNA sequences show little change from the parental sequence, suggesting that either the dRNAs were recently generated, or that selection does act upon the dRNAs (Batuman et al., 2010). Indeed, the conservation (Knorr et al., 1991; Graves et al., 1996) and repair of mutant dRNAs (Kim et al., 1993) has been observed, indicating the latter situation is most probable. In the absence of a non-CTV descendant of the hypothetical novel closterovirus, or discovery of strongly divergent dRNAs in citrus, the recombinant origin of the asymmetrical 5′ and 3′ halves cannot be conclusively proven.

The alternative is that there was a single proto-CTV strain whose genes have evolved under differential selective pressure (Mawassi et al., 1996; Karasev, 2000), both within the genome, and between strains, over evolutionary time. That selective pressures are not equal across the genome can be inferred from the FEL and MEME analysis in this study, in which it was found that the percentage of negatively selected sites varied from 19.6 to 54.8% per ORF, and positively selected sites from 0 to 1.9%. Interestingly, the most diverse region of CTV, ORF1a is also under very strong negative selection, with 47.0% of residues under negative selection; similar results were reported by Martin et al. (2009), although current purifying selection can mask episodes of ancestral positive selection (Murrell et al., 2012). The strength of selection is also not consistent within a single gene, for an examination of ORF1a found that, each of the functional domains showed a higher percentage of negatively selected residues and conversely fewer positively selected residues than the inter-domain regions. These selective pressures correlated with an average of 90.8% sequence identity within domains, as opposed to 81.9% in the inter-domain regions.

It has been remarked upon previously (Albiach-Marti et al., 2000; Silva et al., 2012) that CTV is an inherently stable virus with a very low rate of nucleotide substitutions, or rate of evolution, estimated to be 1.73 × 10^−5^ nucleotide changes, per site, per year based on coat protein sequences (Silva et al., 2012). In this study we observed a rate of 6.67 × 10^−5^ changes, per site, per year, although only changes fixed in the population across the entire genome were considered, leading to a possible underestimation of the rate. The low nucleotide substitution rate of CTV may be due to linear rather than exponential replication (Silva et al., 2012), loss of fitness in mutants, or due to population size, in which small populations evolve faster than larger populations (Sanjuan, 2012). However, estimating substitution rates assumes a constant rate of evolution, whereas a population may evolve rapidly when confronted with a changing landscape of selective conditions, such as movement into new areas, hosts, or vector systems (Nichol et al., 1993; Holland and Domingo, 1998; Moya et al., 2000).

Higher rates of evolution within specific regions of viral genomes have been observed in the E1/E2 genome region of HCV (Gray et al., 2011), the HA1 domain of *Influenza A virus* (Bhatt et al., 2011), and the coat and HAM1h proteins of *Ugandan cassava brown streak virus* (Mbanzibwa et al., 2011); critically, the rapidly evolving regions are involved in host-pathogen interaction or defense responses, indicating the importance of external factors on evolutionary rates, which will be discussed in the following section.

Did ORF1a and, to a lesser extent, other regions of CTV rapidly diversify in the past, taking separate paths across the adaptive landscape? Evidence from MEME and FEL analysis suggests that this was the case, although it is likely that there was a significant difference in rate between lineages. Differences in evolutionary rate between genotypes of the same species has been observed in the E1/E2 and NS5a genes of HCV subtypes 1a and 1b (Gray et al., 2011), within a beta-barrel epitope of the envelope of *Japanese encephalitis virus* (Murrell et al., 2012) and the coat protein of subgroups 2a, 3a, and 3b of CMV (Moury, 2004). For CTV, MEME analysis found episodic diversifying selection in most ORFs, with the exception of p6. Four genes, p23, p61, ORF1a, and ORF1b had multiple positive selection events in lineages leading to the extant CTV strains. The first two genes are respectively responsible for suppression of silencing, as well as controlling negative strand accumulation (Satyanarayana et al., 2002; Lu et al., 2004), and virion assembly (Satyanarayana et al., 2000) respectively, while the latter two are necessary for replication (Satyanarayana et al., 1999). The diversification of p23 is to be expected, as both host antiviral RNAi genes and viral suppressors of silencing are known to rapidly evolve (Obbard et al., 2009), and strain-specific mutations may be the result of adaptation to specific hosts. The p61 protein is a HSP90-type molecular chaperone, involved in CTV virion assembly (Satyanarayana et al., 2000) and, in other viruses, RNA recruitment and assembly of the viral replication complex (Huang et al., 2012). Plant homologs of p61 have also been implicated in assembling RNA-induced silencing complexes with AGO1 (Iki et al., 2010), therefore strain-specific diversification of CTV p61 may be involved in host-interaction or as a pathogenicity factor. The replication components, ORF1a and 1b have evolved under strong host-specific selection, as they interact with co-opted host RNA-binding proteins and molecular chaperones to form a viral replication complex (Huang et al., 2012; Mine and Okuno, 2012). In addition, the helicase domain of the *Tobacco mosaic virus* (TMV) replicase protein has been found to bind to the host NAC-domain transcription factor, suppressing host defense responses (Wang et al., 2009), suggesting that replication associated proteins have multiple functions, hence multiple selective pressures acting upon them, and this shifting balance will move a sequence across the adaptive landscape.

To summarize, it is possible that CTV evolved through multiple introductions of one or several proto-closteroviruses in citrus and subsequently recombined. Unfortunately this remains hypothetical in the absence of a non-CTV closterovirus descended from one of these proto-closteroviruses. Recombination with a dRNA is also possible, although little is known about how much variation a dRNA can develop whilst still retaining the major functional domains. It is more likely that the divergence observed in ORF1a is the result of an adaptive radiation in different proto-citrus hosts, with a variable evolutionary rate within and between strains. The extent of the divergence differs between the 5′ and 3′ halves of the genome, which is due in part to extensive recombination, discussed later, and to competing selective pressures of adaptation to new host species and new selective peaks, whilst retaining multiple biological functions within and between domains.

### Promiscuous recombination

Recombination is a significant factor in CTV evolution (Martin et al., 2009), producing variants with potentially different properties to the parental isolates (Sztuba-Solinska et al., 2011), and as mentioned earlier, allowing a shift of evolutionary trajectory. To continue the adaptive landscape metaphor, recombination allows a population to leap from fitness peak to peak if selectively favored, but if not it can be akin to jumping off a cliff, leading to extinction of that genotype. Recombinants have long been known to be a factor in the emergence of new CTV strains; one of the earliest genomes to be sequenced, SY568 from California (Yang et al., 1999) is a known recombinant, from a mixed population (Vives et al., 1999, 2005), as are B165 (Roy and Brlansky, 2010), Kpg3 (Biswas et al., 2012), all members of the RB (Harper et al., 2010; this study), and T68 genotypes (this study). Recombinants readily occur in mixed infections of CTV strains (Rubio et al., 2001; Scott et al., 2013), which raises the question of why, if recombination can repair defective sequences (Rao and Hall, 1993; Borja et al., 1999), and allow a rapid change in fitness (Sztuba-Solinska et al., 2011) and evolutionary trajectory, recombinants are not found between all CTV strains, and in all regions?

The probability of generating a viable recombinant depends on both viral and host factors. First, it requires that both parental strains be present in the same host, and infect the same cell (Sztuba-Solinska et al., 2011). The recombinant must then be able to replicate and establish a systemic infection. Evidence in this study indicates that there is strain-specific co-evolution in functional domains of ORF1a/1b and, although not investigated, potentially other parts of the genome. Furthermore, the majority of CTV recombinants identified are between isolates of more closely related strains, for example in Kpg3 and NZ-M16 between VT and T3, in NZ-B18 and B165 between T68 and VT, and in isolates NZRB-M17 and TH30 between RB and T36 (Figure 4). Recombination events between more diverse strains were rare, the insertion of a VT-like p65 ORF into RB is one example although, as the rest of the 3′ half of that strain is T30-like, it may not be as drastic a change. One may suggest that co-evolution of functional domains within strains is a limitation on which genotypes may form viable recombinants *in vivo* that if not lethal, may at least reduce fitness and prevent the recombinant from becoming fixed in the population. The exception is the complete replacement of the 5′ or 3′ half of the genome, an event that as noted earlier, has produced the RB and T68 strains, as well as isolates NZ-M16, HA16-5, and SY568 (Vives et al., 1999). It may be proposed that complete replacement of the 5′ half avoids a reduction in fitness as all components necessary for replication are replaced en bloc.

The sites at which recombination can occur may be limited to specific hotspots, sites where recombination frequently occurs (Sztuba-Solinska et al., 2011). Such sites have been observed in PPV (Glasa et al., 2002), *Watermelon bud necrosis virus* (Kumar et al., 2010), and *Brome mosaic virus* (Olsthoorn et al., 2002; Shapka and Nagy, 2004); it may be proposed that there is such a site within the CTV region containing ORF1b-p33 (Vives et al., 1999, 2005; Hilf, 2010), although unlike in the aforementioned examples no features that would promote recombination, either stem-and-loop secondary structures (Glasa et al., 2002; Kumar et al., 2010) or AU-rich regions (Shapka and Nagy, 2004) have been identified in CTV at this site (Vives et al., 1999, 2005; Harper unpublished), or surrounding the p65 recombination site present in the RB strain (Harper et al., 2010).

Finally, if viable, the recombinant faces competition with, and selection against, other CTV isolates in the population; this is of particular importance as CTV isolates have been shown to exclude super-infection by closely related sequences (Folimonova et al., 2010). At time of writing one region involved in this response has been identified: the absence of homologous p33 sequence is necessary for super-infection of one isolate by another (Folimonova, 2012). If super-infection exclusion of a newly generated recombinant does occur, this reduces the probability it will become fixed in the population, or be acquired by an aphid vector and transmitted to a new host, thus in all likelihood leading to extinction.

### The selective landscape

Having established that each gene is evolving under differential selection pressure, and at a different rate, what factors may be at play in determining the topography of the adaptive landscape over which the CTV genotypes have evolved and diversified? We have already mentioned the powerful effect of host-adaptation on the evolution of specific CTV proteins to permit replication and systemic infection, yet little has been said about citrus itself, for diversification in the host is paralleled by diversification in the pathogen. Indeed, host range diversification may be proposed to be a necessary precondition for strain divergence, for two other plant viruses with recognized strain diversification, PPV and CMV, also exhibit significant host diversification, the former infecting many *Prunus* species (Candresse and Cambra, 2006), whilst the latter infects over 1000 herbaceous, shrub, and tree species (Roossinck, 2001). This is not true of all viruses however, for TMV infects species from 30 different families, yet shows little segregation into strains (Kearney et al., 1999); it is possible that the evolution and diversification of viruses into strains differs markedly between those infecting annual hosts that are removed or die at the end of a growing season, and perennial species in which an infection can persist for decades.

The host range of CTV is limited to members of the *Rutaceae*, with the exception of few non-Rutaceous *Passiflora* species (Moreno et al., 2008). All species of the genus *Citrus*, including the commercially important sweet and sour oranges, limes, grapefruit, lemons, and mandarins are susceptible to CTV to some degree (Muller and Garnsey, 1984; Moreno et al., 2008), as are members of the related genera *Microcitrus*, *Clausena*, *Eremocitrus*, *Aegle*, *Aeglopsis*, *Afraegle*, *Citropsis*, *Severinia*, *Swinglea*, and *Atalantia* (Muller and Garnsey, 1984; Yoshida, 1996), although the last three demonstrate some degree of resistance to the virus (Muller and Garnsey, 1984; Garnsey et al., 1987; Mestre et al., 1997), as do *Fortunella crassifolia* and *Poncirus trifoliata* (Mestre et al., 1997). Such a range of host species creates a bewildering array of potential selective factors, peaks, and valleys across the landscape. Each species will differ to some degree in physiology, gene expression, metabolism, and antiviral defenses, and an isolate at an adaptive peak in one host may be less fit in another. For example, CTV isolate T36 has been shown, through use of a GFP-expressing clone, to readily infect *C. macrophylla*, yet have a decreasing gradient of cells infected in *C. sinensis* and *C. paradisi*, to a few scattered cells in *C. aurantii* (Folimonova et al., 2008), which would suggest that T36 has a minimum capacity for replication and movement in *C. aurantii*. Curiously, it has also been found that different combinations of three genes, p33, p13, and p18, are dispensable for infection of *C. macrophylla*, *C. aurantifolia*, *C. sinensis*, *C. paradisi*, *C. micrantha*, *C. latifolia*, and *C. medica* by a T36 clone (Tatineni et al., 2011), while *C. aurantii* requires the genome to be intact, suggesting that each host species exhibits variable selective pressure on different regions of the CTV genome.

There are also differences in virulence between strains, for example T36 isolates can readily infect *C. maxima* cv. “Red Shaddock” pummelo, yet members of the VT and T30 strains take much longer to produce a detectable infection; suggesting a form of resistance in this cultivar (Hilf, 2005). Differential reactions to CTV strains have also been observed in *P. trifoliata* (Harper et al., 2010) and *C. maxima* (Garnsey et al., 1996) suggesting that host-specificity has contributed to the diversity of strains observed today. Furthermore, it may be proposed that resistance genes have contributed to the emergence of the resistance-breaking or “RB” strain of CTV that can systemically infect *P. trifoliata* (Harper et al., 2010), where the resistant host provides a refuge free of competition from other strains, and a potential reservoir of inoculum to spread to other trees. This is most clearly illustrated with soybean in which three resistance loci against *Soybean mosaic virus* (SMV) exist in different cultivars (Chowda-Reddy et al., 2011), which has led to the evolution of specific strains capable of overcoming a single loci, yet no “super strain” has emerged capable of overcoming all loci at once, as this requires multiple concerted mutation of the CI, HcPro, and P3 genes (Chowda-Reddy et al., 2011). Similar limitations in citrus hosts likely account for no characterized strain being capable of infecting all potential hosts equally.

It has been mentioned earlier that host defenses play a significant role in determining the topography of the adaptive landscape, for example resistance genes in *P. trifoliata* restricting virus movement, whilst selecting for mutants better able to replicate and systemically infect host species, such as the CTV resistance-breaking strain “RB” which can systemically infect *P. trifoliata* (Harper et al., 2010). One host defense mechanism, RNA interference, targets the viral genome for degradation via both host- and pathogen-derived small interfering RNAs (Dunoyer and Voinnet, 2005). Differences in host cellular siRNAs have been proposed to determine whether specific tissues are permissive of viral infection (Dunoyer and Voinnet, 2005). The strength of selection exerted by RNAi on viruses is illustrated by the prevalence of virally encoded suppressors of silencing, which are found in potexviruses, potyviruses, cucumoviruses, and closteroviruses (Dolja et al., 2006). CTV encodes three suppressors of silencing: p25, p20, and p23 (Lu et al., 2004), of which the latter two were observed in this study to show significant variation between strains, and frequent episodic diversifying selection, suggesting that there is constant adaptation to changes in the siRNA complex within and between hosts, in effect, an “arms race” (Obbard et al., 2009). Silencing itself can affect the evolution of viral genomes in two ways, by selecting for “escape” mutations that alter the target sequence and prevent recognition and degradation (Leonard et al., 2008), or by selecting for nucleotide compositional changes in the viral genome to match host mRNAs (Dunoyer and Voinnet, 2005); the latter has been observed in CTV (Cheng et al., 2012) and may be a significant genetic barrier to the divergence of CTV strains, and also a potential explanation for the absence of intermediate sequences between the major lineages, as an isolate cannot occupy all possible permutations of sequence space (Roossinck and Schneider, 2006; Domingo et al., 2012).

Finally, in the absence of human intervention, the only means by which CTV is transmitted is by aphid species (Roistacher and Moreno, 1990). The aphid vector species exerts selective pressure on CTV isolates by selectively transmitting some isolates or strains rather than others, for example T3 was transmitted by rates of between 19 and 30%, using *Aphis gossypii* (Bar-Joseph et al., 1977), while NZ-M16, a member of the same genotype was unable to be transmitted by *T. citricida* (Harper et al., 2009). Similarly, *A. gossypii* was capable of transmitting isolates of the VT strain whereas *Toxoptera aurantii* and *A. spiraecola* could not (Raccah et al., 1976). These data suggest co-evolution with specific vector species, likely those prevalent in the region, and those that feed on the host species, in which the strain originated. The same vector species will also transmit strains or isolates at different rates, suggestive of strain-specific co-evolution, for example Raccah et al. (1980) reported rates of transmission for a series of Israeli isolates of between 5.6 and 37.5% with *A. gossypii*, while Broadbent et al. (1996) reported transmission rates of between 5 and 55% with *T. citricida* in Australia. Aphid transmission is particularly important for the evolution of new or novel variants of a strain, for as mentioned earlier, weakly negative, neutral, or even positively selected variants or recombinants may not reach fixation, reducing the probability of transmission, without which it will become extinct with the death of the host. Aphid transmission also acts as a bottleneck, removing a proportion of the quasispecies from the source plant, to a new host where it may evolve in a different direction from the original population (Domingo et al., 2012). The T36 isolate, originally extracted by aphid transmission from a severely declining field tree in Florida in 1975 (S. Garnsey, personal communication) is an example of this phenomenon for it is less pathogenic than most T36 strain field isolates, which could be considered a neutral or positively selected trait, yet it is also very poorly transmitted by aphid species compared to other isolates of the strain (<1 versus 40–50% success rate) (Harper, unpublished). The separation of this otherwise negatively selected mutant from the original population eliminated much of parental quasispecies and allowed a different evolutionary path to be taken, such that today there is little probability that the original phenotype would be restored, as T36 and highly transmitted field isolates FS577, 701 and 703 differ by 35 non-synonymous substitutions spread across the genome, a significant genetic barrier.

In summary, the adaptive landscape over which CTV strains have evolved and diversified is comprised of host factors, including species, resistance genes, and active host defenses such as RNAi. Vector species also exert significant selection on specific strains, and are important for the persistence and spread of novel variation.

### Strain classification and diagnosis

As mentioned earlier, CTV isolates have been classified and grouped by their phenotype, virulence, host range, serology, and more recently, using sequence homology of one or more genes (Moreno et al., 2008). Unfortunately, there has been no concerted effort to classify what constitutes a strain, leading to a proliferation of newly sequenced isolates being referred to as new strains with little justification. In addition, the link between genotype and phenotype is also unclear, and while the role of several genes in phenotypic expression has been indicated (Fagoaga et al., 2006; Albiach-Marti et al., 2010; Tatineni and Dawson, 2012), how often minor differences in sequence alter pathogenicity has not, therefore classification based on phenotype such as “stem pitting” or “seedling yellows” strains, or “severe” and “mild” strains is ill-advised.

Complicating matters is a lack of consistency in choosing a region or regions to analyze, with CP, ORF1a/b, and sundry 3′ genes all being targeted in different assays (Hilf et al., 2005; Nolasco et al., 2009; Roy et al., 2010). Diagnosis with the coat protein alone is a historical legacy, as it is the most highly conserved and least variable of the 3′ genes with 93.4% nucleotide identity and 96.3% amino acid identity between isolates examined in this study. Despite a suggestion that its conservation renders any mutation significant (Nolasco et al., 2009), the CP is not reflective of the complete genome and can in no way explain the divergent 5′ and 3′ halves of CTV strains, nor extensive recombination. For example the CP phylogeny of isolates examined in this study groups some T68-like isolates with VT, and splits the Asian VT subgroup, whilst grouping Kpg3, HA16-5, and HA18-1 together (data not shown). The other 3′ genes show differing levels of conservation and variation between strains, and while they may be appropriate for distinguishing one strain from the rest, we have identified no ORFs from which all six extant strains may be distinguished. In contrast, ORF1a/1b is the most suitable region for phylogenetic reconstruction, as divergence between strains, such as between VT and T3, is most apparent in the 5′ end of the genome (Hilf et al., 1999; Albiach-Marti et al., 2000), and contains conserved functional domains (L1, L2, HEL, and MET) that show strain-specific motifs and hence are suitable sites for primer design. Ideally, one would require the complete genome to be amplified to make an accurate diagnosis of strain type and to identify any potential recombinant regions, such as by using small RNA sequencing as in this study, although given the prevalence of mixtures in the field this is neither practical nor cost-effective for large-scale surveys. We do however suggest that future diagnostic assays be designed to (a) amplify multiple sites within both ORF1a/1b, given the frequent recombination, and (b) design specific primers or probes for each strain at the same site to correctly identify potential recombinants.

In this study we have described six strains of CTV named T36, VT, T3, RB, T68, and T30, defined by separation of their complete genome phylogenies, and distance between groups. We further propose, based on genome phylogeny and recombination analysis, that the type isolates for each strain be assigned as follows: T36: isolate T36 (U16304), T3: isolate T3 (KC525952), T30: isolate T30 (AY260651), RB: isolate NZRB-TH28 (FJ525433), and T68: isolate T68-1 (JQ965169). Due to the bifurcation of the VT genotype, we propose that while not divergent enough to separate into novel genotypes, the Asian and Western subtypes of VT be recognized with the type isolates T318A (DQ151548) and FS701 (KC517494) respectively. Isolate HA16-5 (GQ454870) on the basis of sequence appears to be a novel strain, through recombinant in origin; until a similar sequence is found, the classification remains tentative.

It is likely, as with many crop species (Roossinck and Schneider, 2006), that the ancestral population of CTV is far more diverse than what is currently known and only a subset are present in commercially produced citrus, therefore we must establish criteria to determine whether a new sequence is either novel, or a member of one of the presently described genotypes. Firstly, we must discourage the assigning of new or novel strains on the basis of partial or fragmentary sequence; the complete genome is required for accurate placement. To be a novel strain, the complete genome sequence should differ by >7.5% at the nucleotide level, the minimum distance between VT and T3, and by >8% at both the nucleotide and amino acid levels in ORF1a, the minimum distance between VT and T30. Finally, a novel strain must be examined for recombination with the type members of the extant strains listed above, whilst being recombinant in origin does not disqualify a sequence from being novel, it should show the nucleotide or amino acid divergence shown above to be classified.

### Concluding remarks

The existence of strains, if defined as distinct phylogenetic groups with a shared ancestry as found in PPV (Candresse and Cambra, 2006) and CMV (Roossinck, 2001), is a rare phenomenon amongst plant viruses and is almost unknown amongst the Closteroviridae. CTV is the exception, with at least six extant strains that exhibit a wide range phenotypic characteristics. These strains may have evolved through either a single introduction into citrus and subsequent radiation, or through multiple introductions followed by recombination; which scenario is more likely is obscured through subsequent evolution over time and the absence of extant proto-closterovirus sequences. Regardless of their origin, CTV strains have evolved and diversified across the adaptive landscape, a topology comprised of many host and vector species, that have exerted variable selective pressure on different parts of the genome, and indeed, between strains leading to diversity within non-functional domains regions, such those within ORF1a for example, while the 3′ genes, which include structural and replication associated proteins, are much more conserved. Functional constraints, together with co-evolution of the replication domains and host-selection pressures on codon choice have acted to decrease the likelihood of moving between adaptive peaks by mutation alone. Recombination, rather than mutation, has been shown to be the major factor in CTV strain evolution, producing three of the six extant strains, although evidence suggests that co-evolution reduces the likelihood of recombination between any two strains. Why then, is an understanding of strain evolution important? Knowledge of the selective pressures and constraints acting upon CTV strains is crucial to the development of cross-protection programs, for the development of infectious clones for field release, and for the breeding of new, resistant citrus cultivars. It is hoped that further research into the link between genotype and phenotype will yield significant advances in citrus production.

## Conflict of Interest Statement

The authors declare that the research was conducted in the absence of any commercial or financial relationships that could be construed as a potential conflict of interest.
